# An examination of immersive journalism by bibliometric analysis from 1999 to 2023

**DOI:** 10.1016/j.heliyon.2024.e34263

**Published:** 2024-07-14

**Authors:** Mehmet Arif Arık, Murad Karaduman, Sibel Karaduman, Çiğdem Karakaya, Fulya Erendağ Sümer, Zuhal Gök Demir

**Affiliations:** a Faculty of Communication, Department of Journalism, Akdeniz University, Antalya, Turkey; b Faculty of Communication, Department of Department of Radio, Television and Cinema, Akdeniz University, Antalya, Turkey; c Faculty of Communication, Department of Public Relations and Publicity, Akdeniz University, Antalya, Turkey

**Keywords:** Immersive journalism, Virtual reality, Bibliometric analysis, 360-Degree video, Narrative journalism

## Abstract

Immersive journalism is an innovative storytelling approach that aims to enable the audience to experience the event or situation in the news using virtual reality, unlike traditional news narration. In this study, the literature related to the subject was searched using the keywords Immersive Journalism, 360-Degree Video, Narrative journalism, Newsgame, VR Storytelling through the Web of Science database and a data set was created from 955 publications between 1999 and 2023. No filter was applied to the studies in the data set of the study and articles, books, and early access publications as well as book chapters, editorial materials or conference proceedings in the Web of Science database were included in the study. Bibliometric analysis techniques were applied to the obtained data set and the collaboration status and citation maps of authors and institutions were revealed together with various parameters including authors, institutions, keywords used, number of citations and distribution of publications according to countries.

As a result of the analyses, while the number of studies related to the subject has increased since 2017, the highest number of studies was conducted in 2021. The most articles on the subject of the study are in the “Communication” category. While the majority of academic studies on the subject were carried out in the United States, studies originating from this country were cited the most. While “Virtual Reality” is the most commonly used word in the studies on the subject, “IEEE Access” stands out as the journal with the most publications on the subject. On the other hand, the study revealed important authors, studies and themes in the literature on immersive journalism, emphasised the interactions between authors and disciplines, and provided a road map for future research. The study has limitations in terms of the publication languages of some of the studies in the dataset, the time period of the dataset and the singularity of the preferred database.

## 1 Introduction

The rapid advancement of technology has transformed storytelling and led to the emergence of digital-based interactive forms. High technologies such as 5G, drones, artificial intelligence, augmented reality and virtual reality, which are added to the news production, distribution and presentation processes in the journalism profession, have led to the emergence of the concept called “Hitech Journalism”, which characterizes new generation journalism activities [[1], [2], [3], [4], [5]] Hitech journalism is defined as the journalistic practices of the future, shaping the next generation of journalism [6], robot journalism [7], the use of interactive algorithms and digital assistants on news websites [8,9], imaging techniques such as drones and 360° cameras [10,11] and immersive journalism using virtual reality or augmented reality [12].

Immersive journalism, which is the subject of the study, is defined as a form of journalism that enables viewers to obtain first-person experiences of the events and phenomena described in the news through cameras that provide 360-degree image recording [[12], [13], [14]]. This new format allows people who want to access the news to experience the news story intensively and feel as if they are in the news position [15].

It is seen that the literature on immersive journalism has been examined on different dates and through different databases with systematic literature review [[16], [17], [18]] or scientometric analysis [19] methods but few have analyzed it using bibliometric methods [20]. As there is a growing interest in immersive technologies in different fields and disciplines, research trends and opportunities in Virtual Reality and Immersive Virtual Environments have been researched bibliometrically such as renewable tourism [21,22] environmental comfort indexed behaviour, Virtual environment applications for occupant comfort, geographical heritage mapping and museum applications [[23], [24], [25]]. These studies show that there is a trend towards immersive technologies to continue location-dependent commercial, educational, and service activities in virtual reality or augmented reality areas in a sustainable, cheap, comfortable and effective way. In addition to the same motives, it is important to perform the journalism profession with immersive technologies in order to increase the impact intensity of the news, to bring the reader closer to the place where the news takes place and to create experience [12,13,26,27].

This study analyses the state of the literature on immersive journalism. Thus, its objective is to emphasize potential topics and areas within the field of journalism that are ready for further investigation and improvement for future research. In the study, a data set was created from the studies in the WoS database and the words “immersive journalism”, “360-Degree Video”, “Narrative Journalism”, “Newsgame” and “Vr Storytelling” in the article titles, abstracts or keywords and bibliometric analysis techniques were applied. With the performance analyses applied to the data set, the types of studies in the literature, authors and the countries of origin of these studies, and with the science mapping techniques, the most cited studies and the co-authorship and citation networks of the studies with the authors were revealed.

Unlike other bibliometric or systematic literature analyses in the field of immersive journalism [[16], [17], [18], [19]], instead of addressing the literature within the framework of a specific research question or making a comparative analysis of the literature in a certain date range, this study was carried out to create a general and inclusive intellectual map by examining certain parameters in the literature.

The rest of the paper is organised as follows. While the first section provides information about Immersive Journalism and VR storytelling the second section introduces the search strategy, database and analysis methods. The third section presents the results of the bibliometric analysis, such as the co-citation map, the distribution of studies by year and the most cited articles. The last section contains the discussion.

### 1.1 Immersive journalism and VR storytelling

Technological developments have led to sharp changes in the production, distribution and consumption practices in the journalism profession [28]. In particular, the use of new media technologies has led to the preparation of more personalised content and the emergence of a more personalised version of journalism [29,30]. While a more automated and robotic production mechanism has become possible thanks to artificial intelligence applications [31], visuality, such as infographics, and interactivity, such as news games [26], have emerged. One of these types is experience-oriented immersive journalism. Immersive journalism is a concept that cannot be considered separately from the concept of virtual reality and virtual reality technologies. Essentially, immersive journalism is a form of journalism made possible by virtual reality and virtual reality technologies.

Virtual reality blocks information from the real world and allows the flow of information about a reality created by digital modelling that can be followed by more than one sense. The fact that virtual reality appeals to the senses of hearing and vision, which are especially important senses for human perception, causes artificial reality to create a real world feeling in the user by creating a high degree of illusion [32]. The sensorimotors of wearable technologies such as VR glasses, headphones and motion tracking gloves increase the sense of reality by tracking head, hand and arm movements [33]. This situation is related to the concept called “immersion”, which is among the features of virtual reality technologies [34]. People immersed in the virtual environment break away from the place where they are located, think as if they are there and perform their actions accordingly [15]. On the other hand, in virtual reality, individuals can interact with both machine and human with various software, hardware and platforms. This interaction is an important condition to increase the belonging of individuals towards virtual reality. Finally, virtual reality combines the worlds of fantasy and imagination with the senses of individuals, making it possible for them to have the reality they always want. This situation causes the perception of virtual reality to be preferred to the reality in the outside world and even blurs the lines between these two realities. As a result of the coexistence of these three features, individuals have the feeling of being in a certain location or experiencing an event or situation [35].

The process that started with Facebook's acquisition of Oculus in 2014 triggered major technology companies to turn to virtual reality (VR) and augmented reality (AR) [32,36]. The commercialisation of virtual reality hardware and software and its widespread use in civilian use has also popularised virtual reality-based storytelling using 360-degree, multiple or moving imaging systems and touchscreen computer or portable device screens. In this popularisation process, it is inevitable that the journalism profession will follow the trend and virtual reality tools will become a mass communication tool [32].

Immersive journalism is a type of journalism in which users can experience news events, places and stories first-hand [12]. The concept was first used by Nony De la Peña et al. [12] to distinguish the type of journalism experienced through virtual reality using an omnidirectional or 360-degree camera from the news game concept.

Immersive journalism is nowadays mostly implemented using 360 news videos [15]. However, when immersive journalism is realised using more sophisticated virtual reality systems, it provides a more intense experience. For example, the virtual reality documentary “Project Syria” created by De la Peña in 2014 to experience the events of the Syrian civil war through the eyes of real people is a good example of the use of these technologies for immersive journalism [32,37].

The fact that experience is the main element in immersive journalism creates controversial situations in terms of journalistic ethics and practices [33,38]. In immersive journalism, even though users are aware that they are in a virtual reality, the intense first-person experience they will experience about the news event may affect similar experiences they will have in the real world. At the same time, how journalists, who are obliged to comply with certain ethical codes such as objectivity in the news production process, will overcome the emotional ties or empathy that users will establish with the event or person subject to the news while preparing content related to immersive journalism poses other question marks.

## 2 Materials and methods

### 2.1 Materials

The Web of Science Core Collection (WoS) database was used to create the database of the study due to its diversity, size, high quality and convenience [39,40]. Furthermore, WoS covers more than twenty thousand bibliometric studies spanning over 250 disciplines [41]. The WoS and Scopus databases have long been extensively compared, although in certain areas they are better than each other. However, the bibliometric community has not concluded that one is generally better than the other [42]. In this direction, the choice of database was limited to WoS since the studies of the required quantity and quality for the research can be accessed from the WoS database. The dataset of the study consists of 955 publications consisting of research articles, early access articles, review articles, books, book chapters, proceedings, editorial materials and book reviews in the WoS database between 1999 and 2023. In determining the keywords for the search, technologies, application and production techniques, terms and concepts related to Immersive journalism were taken into consideration [12,13,17,19,26,32,33]. During the search for the dataset, the conjunctions “AND” and “OR” were used and the keywords immersive journalism AND 360-Degree Video (should-search within topic) AND Narrative Journalism (should-search within topic) AND Newsgame (should-search within topic) AND Vr Storytelling (should-search within topic) were searched in the article title (TOPIC). During the search, the “AND” operator was used to find studies with two or more keywords used together, while the “OR” operator was used to find studies with any two or more words. The phrase “should-search within topic” added during the search was added as a result of performing a quick keyword search between operators. After the noisy data were extracted, analyses were performed using VOSviewer (version 1.6.19), a free program. VOSviewer is a programme that allows bibliometric maps to be examined in full detail, making it easier to examine data with features such as zooming, panning and searching [43].

Statistical qualitative methods such as citation analysis, co-occurrence of keywords, co-citation analysis or co-authorship analysis, which are bibliometric analysis techniques [44] were used to examine the formal characteristics of the literature considered within the scope of the study.

### 2.2 Methods

Bibliometric analysis was chosen as the study method. The bibliometric analysis method is based on objective findings and quantitative techniques, independent of the possibility of the researcher in qualitative research to direct the findings [39,45]. Bibliometric analysis is carried out with performance analyses and science mapping analyses applied to the data set [39]. Performance analysis is based on examining the effectiveness of research components such as researchers, institutions, countries or resources in the literature, which are included in all or part of the literature on the subject under consideration [39,46]. Science mapping, on the other hand, focuses on the interactions, citation networks and collaborations between research components. This study aims to reveal the intellectual structure of the literature on immersive journalism, the performance status and the collaborative relationships between the variables in the literature. Accordingly, the study area was analyzed in line with the following questions.

• Between which years were the articles published?
• In which categories are the articles placed?
• In which index are the articles published?
• What are the countries of origin of the authors?
• What are the main keywords used in the studies?
• What is the level of co-operation and citation status between the authors of the article?

Bibliometric analysis can analyse the structure of scientific knowledge, the rules and forms of diffusion and distribution, and can also present the results through a map of scientific knowledge [47] It can also mine information such as emerging trends, popularity of studies, hotspots and landmark references [48,49].

### 2.3 Performance analyses

**Fig. 1:**
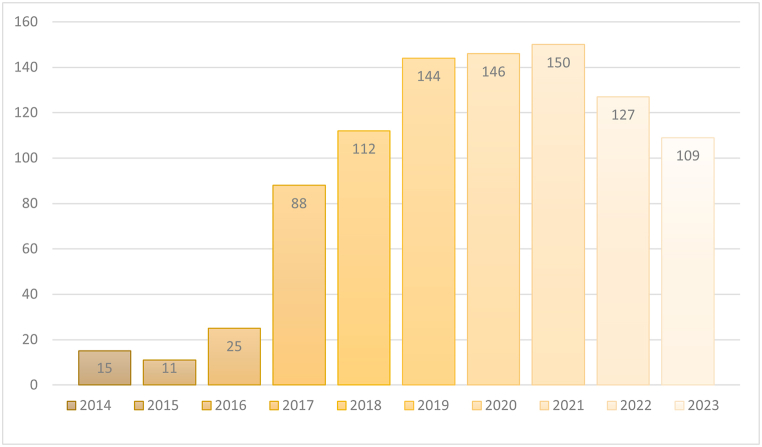
Distribution of publications by years.

Studies on the subject were conducted between 1999 and 2023. However, the period from 1999 to 2009 was limited to 11 publications in total, and maximum 2 publications were made in 2005 and 2006. In 2000 and 2001, no related study was published. Only 19 studies were conducted between 2010 and 2013 and the highest number of studies was conducted in 2007 with 7 studies. The steady increase in the studies on the subject started in 2014 and reached its highest level in 2021 (Fig. 1.). Technological investments and developments, especially in the period between 2015 and 2017, have led to technologies such as virtual reality (VR), augmented reality (AR) and 360-degree video becoming more useful and accessible. On the other hand, the fact that journalists looking for new and effective storytelling methods and academics working in this field started to be interested in these technologies, which offer readers the opportunity to experience events in more depth by immersing them in the story, provides clues as to why studies on the subject showed a great increase between 2017 and 2021.

After 2021, there is a decrease in the number of studies. In this period, the saturation of the field and the shift of the motivation of academicians towards the Covid-19 pandemic, which took place between these years, can be shown as the reason for the decrease in the number of studies on the subject. In addition to this, journalism's dependence on technological tools in news production and distribution processes makes it one of the sectors and professions most affected by technology. In this direction, developments and trends in technology, especially artificial intelligence, create new areas of expertise and study. A shift in academic interest towards artificial intelligence and similar technologically orientated fields is likely for the post-2021 pattern. Data for 2023 shows the lowest number of studies in the last 5 years. However, considering the commercial investments of multinational companies in artificial intelligence and high-tech wearable technologies, it is thought that studies in this field will trend again.

**Fig. 2:**
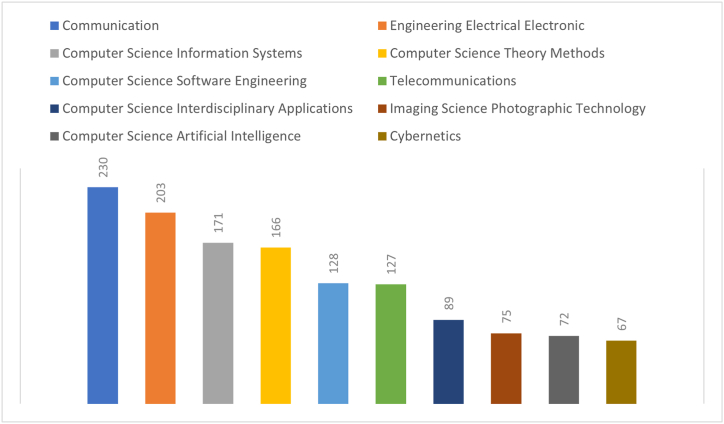
Distribution according to WoS categories.

Since the determined subject has a multidisciplinary structure, it is important to examine in which WoS category the studies carried out in this field are located in order to reveal to what extent which discipline is interested in the subject and to what extent. In this direction, the categories of the analyzed studies are shown in Fig. 2. Communication sciences are in the first place since the subject of the study is directly related to the field of journalism. On the other hand, the categories related to computer engineering constitute the remaining part of the list due to the structure of topics such as 360 imaging, virtual and augmented reality, which are embedded in computer technologies. In fact, the only category that works on the subject and is included in social sciences is communication sciences.

The indexes in which the studies on the subject are included are shown in Table 1. The majority of the studies consist of conference proceedings. On the other hand, the reason why the studies are concentrated in Emerging Sources Citation Index (ESCI) and Science Citation Index Expanded (SCI-EXPANDED) indexes can be shown that engineering disciplines are intensively interested in the subject. The 4th place in the Social Sciences Citation Index (SSCI) is due to the studies related to communication sciences. Apart from book chapters, Arts & Humanities Citation Index (A&HCI) index is also included in the list (see Table 2).

**Table 1:** Distribution of publications according to indexes.

Sequence	Web of Science Index	Record Count	% of 955
1	Conference Proceedings Citation Index - (Science (CPCI–S)	368	38.534 %
2	Emerging Sources Citation Index (ESCI)	221	23.141 %
3	Science Citation Index Expanded (SCI-EXPANDED)	182	19.058 %
4	Social Sciences Citation Index (SSCI)	166	17.382 %
5	Conference Proceedings Citation Index - Social Science & Humanities (CPCI-SSH)	36	3.770 %
6	Arts & Humanities Citation Index (A&HCI)	26	2.723 %
7	Book Citation Index- Social Sciences & Humanities (BKCI-SSH)	20	2.094 %
8	Book Citation Index - Science (BKCI–S)	5	0.524 %

**Table 2:** Top 10 authors with the most publications on the subject.

SEQUENCE	AUTHOR	INSTITUTION	COUNTRY	NUMBER OF PUBLICATIONS
1	Yao Liu	State University of New York	USA	22
2	Cong Thang Truong	University of Aizu	PRC	13
3	Yao Wang	New York University	USA	13
4	Zhi Liu	North China University of Technology	PRC	12
5	Sara Pérez-Seijo	Universidade de Santiago de Compostela	SPAIN	12
6	Duc Nguyen	University of Aizu	JAPAN	11
7	Mengmeng Zhang	North China University of Technology	PRC	10
8	Chao Zhou	National University of Singapore	SINGAPORE	10
9	Jacob Chakareski	New Jersey Institute Technology	USA	9
10	Eun-Seok Ryu	Sungkyunkwan University	SOUTH KOREA	9

The most published authors on the subject are shown in Table 3. When the fields of study of these authors are analyzed, it is seen that they mostly work in the fields of computer engineering, imaging sciences and engineering and telecommunication technologies (Zhou, Ryu, Nguyen, Yao Liu, Thang, Wang, Zhi Liu). In the field of communication and information sciences, only one author (Sara**)** was included in the list. The main reason for this situation is that the topic is directly related to the mentioned engineering sciences and the authors working on this subject have high collaboration rates with each other.

**Table 3:** The top ten most cited articles on the subject.

Sequence	Article	Citation Count	Annual average number of citations
1	Slater, M., & Sanchez-Vives, M. V. (2016). Enhancing our lives with immersive virtual reality. *Frontiers in Robotics and AI*, *3*, 74 [27].	688	74.22
2	De la Peña, N., Weil, P., Llobera, J., Spanlang, B., Friedman, D., Sanchez-Vives, M. V., & Slater, M. (2010). Immersive journalism: Immersive virtual reality for the first-person experience of news. *Presence*, *19*(4), 291–301 [12].	295	19.67
3	Corbillon, X., Simon, G., Devlic, A., & Chakareski, J. (2017, May). Viewport-adaptive navigable 360-degree video delivery. In *2017 IEEE international conference on communications (ICC)* (pp. 1–7). IEEE [50].	217	27.13
4	Corbillon, X., De Simone, F., & Simon, G. (2017, June). 360-degree video head movement dataset. In *Proceedings of the* 8th *ACM on Multimedia Systems Conference* (pp. 199–204) [50]	174	21.75
5	Xie, L., Xu, Z., Ban, Y., Zhang, X., & Guo, Z. (2017, October). 360probdash: Improving qoe of 360 video streaming using tile-based Http adaptive streaming. In *Proceedings of the* 25th *ACM international conference on Multimedia* (pp. 315–323) [51].	170	21.25
6	Qian, F., Han, B., Xiao, Q., & Gopalakrishnan, V. (2018, October). Flare: Practical viewport-adaptive 360-degree video streaming for mobile devices. In *Proceedings of the* 24th *Annual International* *Conference on Mobile Computing and Networking* (pp. 99–114) [52].	161	23
7	Graf, M., Timmerer, C., & Mueller, C. (2017, June). Towards bandwidth efficient adaptive streaming of omnidirectional video over http Design implementation and evaluation. In *Proceedings of the* 8th *ACM on Multimedia Systems Conference* (pp. 261–271) [53].	154	19.25
8	Shin, D., & Biocca, F. (2018). Exploring immersive experience in journalism. *New media & society*, *20*(8), 2800–2823 [54].	152	21.71
9	Bao, Y., Wu, H., Zhang, T., Ramli, A. A., & Liu, X. (2016, December). Shooting a moving target: Motion-prediction-based transmission for 360-degree videos. In *2016 IEEE International Conference on Big Data (Big Data)* (pp. 1161–1170). IEEE [55].	139	15.44
10	Sundar, S. S., Kang, J., & Oprean, D. (2017). Being there in the midst of the story: How immersive journalism affects our perceptions and cognitions. *Cyberpsychology, behaviour, and social networking*, *20*(11), 672–682 [33].	121	15.13

The 10 most cited studies on the subject are shown in Table 4. The first article in the table, “Enhancing our lives with immersive virtual reality”, was published by Mel Slater, who works in the field of virtual reality, and Maria V. Sanchez-Vives, who works in the fields of neuroscience and virtual reality. It is assumed to have a high number of citations in its field, mainly due to the fact that it is a pioneering study in which descriptive concepts on virtual reality are conveyed. In second place is the publication on immersive journalism by journalist and researcher Nonny de la Peña et al. The fact that it is a pioneering study, especially because it offers an innovative method in the interactive transmission of news narratives, explains the high number of citations it has received. The third ranked article ‘Viewport-adaptive navigable 360-degree video delivery’ by Xavier Corbillon, Gwendal Simon, Alisa Devlic and Jacob Chakareski presents proposals, tools and innovative ideas for more efficient display of images from 360-degree cameras on head-mounted displays. The article is considered to be highly cited as it plays a role in the development of an important technical tool on the subject. On the other hand, the studies published by Frank Biocca, Donghee Shin, Shyam Sundar, Jin Kang, Danielle Oprean, who work in the fields of communication sciences, virtual reality and media technologies, are related to immersive journalism. Other articles in the list consist of studies published on the use of 360 imaging technologies, content preparation and innovative technologies in this field.

**Table 4:** Most cited authors, number of citations and link strength.

Sequence	Author	Number of Articles	Citation Count	Connection Strength
1	Slater, Mel	7	1029	4
2	Sanchez-vives, Maria V.	2	963	0
3	Simon, Gwendal	7	447	307
4	Corbillon, Xavier	6	447	299
5	De la pena, Nonny	3	306	4
6	Friedman, Doron	1	295	0
7	Giannopoulos, Elias	1	295	0
8	Ilobera, Joan	1	295	0
9	Pomes, Ausias	1	295	0
10	Spanlang, Bernhard	1	295	0
11	Weil, Peggy	1	295	0
12	Chakareski, Jacob	9	288	115
13	Guo, Zongming	8	276	543
14	Zhang, Xinggong	8	276	543
15	Devlic, Alisa	4	257	60

The most cited authors on the subject are shown in Table 5. When the table is analyzed, it is seen that Mel Slater and Maria V. Sanchez-vives, the authors of the most cited article, are also the most cited authors related to the subject. Gwendal Simon, Xavier Corbillon and Jacob Chakareski, on the other hand, have a high number of publications and citations compared to the average of the table as authors who collaborate intensively with each other. They are also among the authors with the highest connection strength. The authors in the section from 8th to 11th place in the table collaborate with each other in the second place in Table 4, “Immersive journalism: Immersive virtual reality for the first-person experience of news”. The most cited authors related to the topic were the authors of the most cited articles related to the topic in Table 4. This situation both plays the role of a confirmatory analysis in terms of the data set and reveals the importance of collaboration between authors.

**Table 5:** Countries with the highest number of publications on the subject.

Sequence	Country	Number of Articles	% 955
1	United States of America	236	24.712 %
2	People's Republic of China	163	17.068 %
3	Spain	125	13.089 %
4	Japan	53	5.550 %
5	England	50	5.236 %
6	Australia	48	5.026 %
7	Germany	45	4.712 %
8	South Korea	42	4.398 %
9	Brazil	30	3.141 %
10	Canada	28	2.932 %
11	Finland	27	2.827 %
12	Netherlands	24	2.513 %
13	France	23	2.408 %
14	Portugal	16	1.675 %
15	Switzerland	16	1.675 %

### 2.4 Co-authorship analysis

**Fig. 3:**
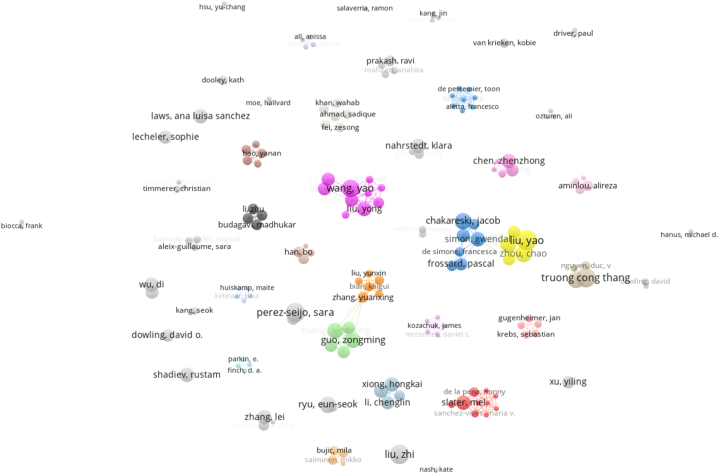
Co-authorship map.

Co-author analysis was performed to reveal the collaboration of the authors. Having at least one document among the 2385 authors in the data set and having at least 50 citations were determined as the cut-off point for the analysis. As a result of the analysis, the map of 168 authors in collaboration is shown in Fig. 3. While the size of the circles in the figure is related to the studies published by the authors, the links between the circles represent the collaboration between the authors. There are more than 12 basic clusters in the map. Authors in the clusters form a collaboration network with other authors in the cluster they are in. In addition, there is a collaboration network between the authors in the blue cluster and the authors in the yellow cluster and between the authors in the green cluster and the authors in the orange cluster. The most intense co-operation and relational strength is in the yellow cluster formed by Liu, Zhou, Chen, Xiao and Li. This is due to the intensive co-operation and co-authorship between the authors in the cluster, especially with Simon in the blue cluster and with other blue cluster authors Frossard, De Simone, Chakareski and Devlic. The second intensive collaboration is between the green cluster formed by Guo, Xu, Xinggong and Xie and the orange cluster formed by Zhang, Bian, Zhao, Liu and Li. In the red cluster, the most cited authors Mel Slater and Sanchez-vives, Maria and de la Pena, Weil, Sanchez, Giannopoulos, Llobera and Friedman are included in the list of the most cited authors. In the purple cluster, there are authors such as Wang and Yuwen with a high number of articles on the subject.

**Fig. 4:**
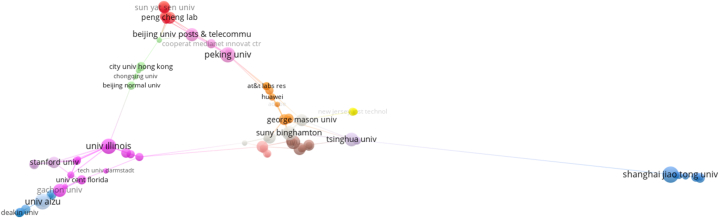
Partner organisation-cooperation map.

In order to reveal the cooperation of the institutions in the field, common institution analysis was applied to the data set. For the analysis, the condition of having at least 2 documents among the 937 institutions included in the article was taken as a cut-off point and the cooperation formed by 284 institutions was mapped (Fig. 4). The size of the circles on the map is related to the number of publications and the connections between the circles are related to the state of cooperation. In this respect, the institutions that created the most publications and documents are Shanghai Jiao Tong University with sixteen publications and Aizu University with fifteen publications. The strongest network of connections and the most intensive co-operation occur within/between the purple and red clusters, where Stanford and Illinois universities are located. The grey cluster with State University of New York (Binghamton) and the pink cluster with Peking University have the second strongest collaboration network. While there are mostly universities among the institutions in the list, there is no Turkish institution in the list.

**Fig. 5:**
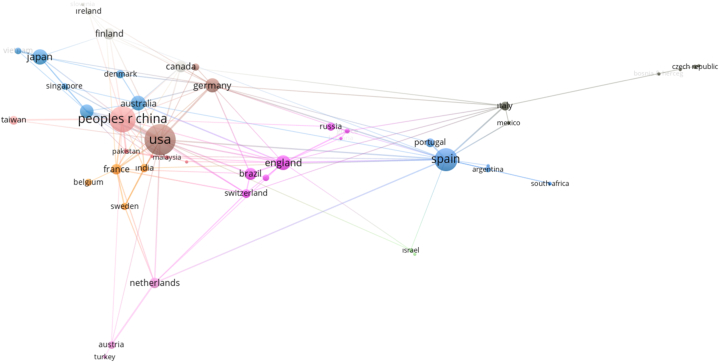
Country co-operation map.

For the country cooperation analysis, no cut-off point was used and all 59 countries with at least 1 document were analyzed. The results of the analysis are shown in Table 5 and Fig. 5. The countries with the highest number of publications on the subject are the United States of America, the People's Republic of China, Spain, Japan and the United Kingdom. These countries are represented by different colour circles in Fig. 5. The most intensive co-operation network and the highest connection power among the countries belong to the USA and these studies received 4026 citations. China, which ranked second, received 1617 citations, while Spain, which ranked third, received 1685 citations. Although the number of citations of China is lower than Spain, China's cooperation network with other countries is stronger than Spain. Another noteworthy situation in Table 5 is the absence of any country from the African continent in the list. It is thought that the main reason for the scarcity of studies on immersive journalism, which is a type of journalism that requires high cost and high technology, is the limited financial situation of countries in the African continent compared to countries in Europe and Asia. On the other hand, the different specialisations of journalists or academics and limited access to regional cooperation and global research networks on the continent may be other factors influencing this outcome.

It is seen that the connection networks in the cooperation map are mostly between geographical neighbours (or countries that exhibit cultural affinity, for example, European countries and Asian countries establishing a cooperation network among themselves).

### 2.5 Citation analysis (CA)

**Fig. 6:**
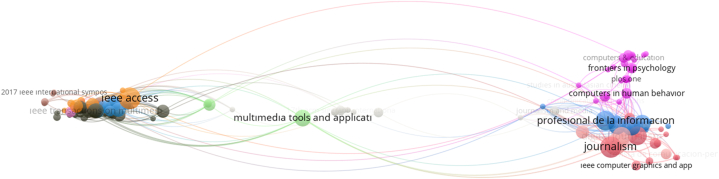
Source citation network.

Citation Analysis applied to the data set revealed the sources used in the publications related to the subject and their relations with each other. In this direction, the situation of having at least 10 citations among 562 sources in total were determined as the cut-off point of the analysis. The source citation map with 167 sources resulting from the cut-off point is shown in Fig. 6. The size of the circles in the figure varies according to the number of studies on the subject. The connections between the circles are related to citation relationships. At this point, the source that contains the most studies with 18 publications is “IEEE Access”, which is included in the Science Citation Index Expanded (SCIE) index and has a journal citation indicator (JCI) of 0.89. Secondly, ‘Journalism’ in the Social Sciences Citation Index (SSCI) and ‘Literary Journalism Studies’ in the Emerging Sources Citation Index (ESCI) have 17 publications each. The journals with more than 10 related publications are “Professional de la informacion”, “Digital journalism”, “Journalism studies",” Revista Lantina de communicacion social” and “Journalism practice”. This situation reveals that journals related to journalism show more interest in publications related to the subject of the study. When the citation status of the sources related to the subject is examined, the journal “Frontiers in robotics an AI”, which is included in the Emerging Sources Citation Index (ESCI) index and received 747 citations, is the journal with the highest number of citations. The journal citation indicator (JCI) of this journal is 0.68. The most cited sources other than Frontiers in robotics an AI are “8th ACM multimedia System Conference (mmsys'17) conference booklet (proceedings) Journalism studies, digital journalism, journalism, “2017 IEEE International conference on communication” conference booklet (proceedings) respectively. The conference proceedings of “8th ACM Multimedia System Conference (mmsys'17)" also has the densest collaboration network and the highest connection strength. As a result of the analysis, the majority of the relevant resources are provided by journals related to journalism.

**Fig. 7:**
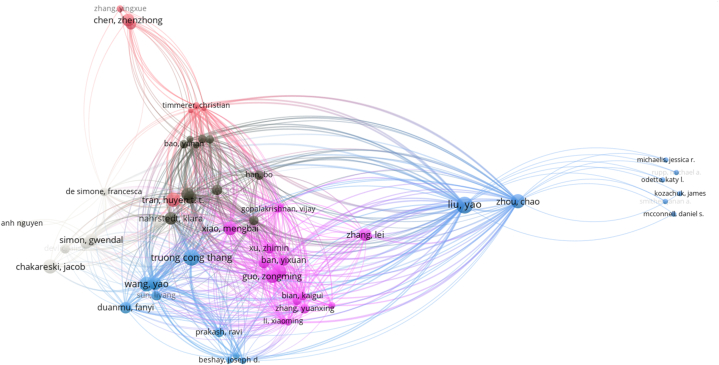
Author citation network.

In order to reveal the citation networks and relationships of the authors, author citation analysis was applied and the status of having at least 100 citations among 2385 cited authors was determined as the cut-off point for the analysis. In Fig. 7, the citation network formed by 73 authors with at least 100 citations are visualised. The clusters in Fig. 7 consist of authors who collaborate among themselves or work in similar disciplines. As a result of the analysis, Yao Liu (202), Chao Zhou (133), Yao Wang (130) and Truong Cong Thang (120) in the blue cluster, Zongming Guo (117) and Mengbal Xiao (134) purple, Huyen Tran (81) in the pink cluster are among the authors with the highest linkage strength. Especially the authors in the center of the map form a citation network not only with the authors in their own cluster but also with the authors in different clusters.

**Fig. 8:**
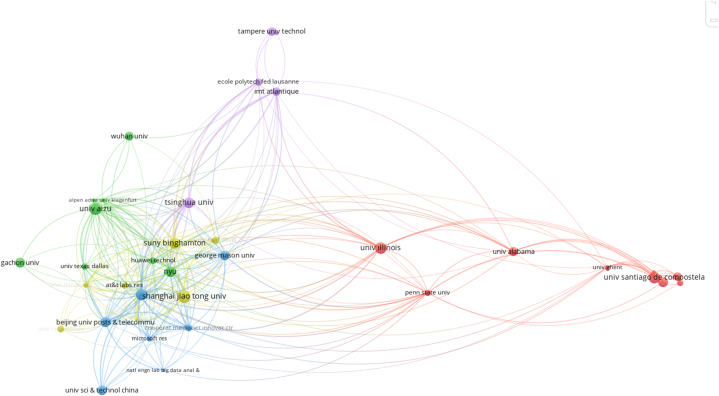
Institution citation analysis.

The analysis was applied in order to reveal the citation relationship between the institutions. Among the 937 institutions in the study dataset, having at least 100 citations was determined as the cut-off point for the analysis. Fig. 8 shows the map created with 44 institutions with at least 100 citations. Almost all of the institutions on the map consist of universities. It is seen that especially geographically neighboring universities or universities with the same cultural affinity form a citation network among each other. Peking (149), State University of New York Binghamton (102), Beijing (92), Shanghai Jiao Tong (86), Tsinghua (75) and George Mason (72) universities are respectively among the institutions on the map in terms of citation link strength.

**Fig. 9:**
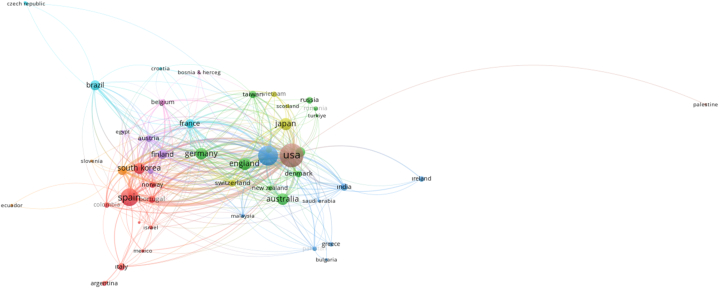
Citation status of countries.

In order to reveal the citation networks and relationships of the countries, 59 countries for which country citation analysis was applied without using a cut-off point are mapped in Fig. 9. The size of the circles on the map is related to the number of studies originating from that country. In this direction, as can be seen in Table 5, the USA, China and Spain are the countries that have carried out the most studies on the subject. The high citation network of academic studies in these countries can be attributed to several complex factors. Countries such as the USA, China and Spain have generally assumed pioneering roles in the field of media and communication, hosted various media institutions and pioneered in this field. This can be seen in the high number of studies on the subject. On the other hand, immersive journalism generally requires the use of new technologies. Technologically advanced countries such as the USA, China and Spain can adapt to such technological innovations faster and may have carried out pioneering research in this field. In addition to this, these countries are generally ranked among the countries with high educational standards.

**Fig. 10:**
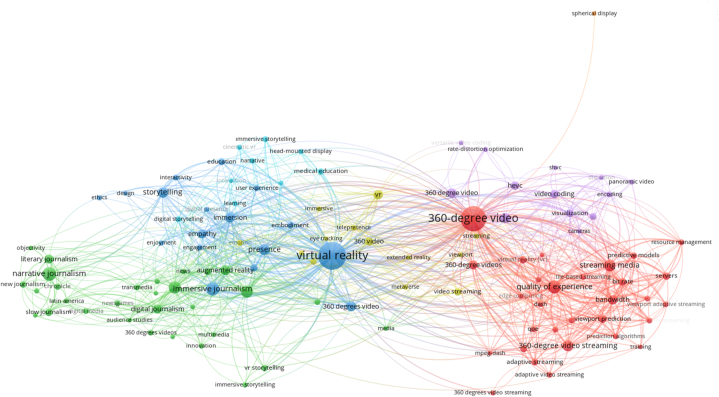
Common usage analysis of keywords.

Word analysis was applied in order to reveal the extent to which the keywords in the studies in the data set were used and the relationships between these words. While there are 2445 keywords in the data set in total, the fact that they were used at least 5 times was determined as the cut-off point for the analysis and the keyword representation map consisting of 113 words are given in Fig. 10. Words that are conceptually or semantically related and close to each other were mostly used together or in a common color cluster. The fact that the keywords appear in different ways in the map is due to the grammar rules of different languages or the different ways of writing the concepts. The most commonly used words in the studies on the subject were “virtual reality” (286), “360-degree video” (278), “immersive journalism (93) and “Narrative Journalism” (56). While the red and purple cluster on the map consists mostly of technical and technological concepts related to computer engineering, the yellow, blue, green and turquoise clusters on the left side of the map consist of concepts used by disciplines such as journalism and cinema.

## 3 Conclusion

Immersive journalism is of great importance for the development and implementation of new generation journalism activities, both in terms of its integrated structure with innovative technologies and as an experience-oriented, more intense and effective form of storytelling.

In this study, all of the studies in the Web of Science index related to immersive journalism, virtual reality and 360 imaging were subjected to bibliometric analysis; it was aimed to reveal the intellectual status, thematic structure, author collaboration and citation interaction trends of the studies in the literature on the subject. In this direction, “955″ studies published between 1999 and 2023 and accepted in terms of compliance with the data set were subjected to analysis, and the leading studies, authors, disciplines, countries and the collaborations and relationships between these variables in terms of citation and publication in the literature were identified and visualised.

In previous studies on the subject, it has been observed that the findings are based on a narrow research question or as a result of the comparison of periodically separated data using the dynamic focus method. This study, on the other hand, differs from other studies as a comprehensive structural situation analysis by revealing the relationships of variables (author, institution or journal) in the literature with the structural focus method. Thus the importance of this study is to show the general variation in the field of immersive journalism by demonstrating the trends in the field.

Firstly, publication number and distribution of studies according to years are analyzed. It is seen that the studies on the subject started to increase steadily in 2017 and after, but this increasing trend turned into a decline after 2021. While there is an increase in the number of studies in parallel with the development of technology related to virtual reality and augmented reality, there is a decrease in the interest of academicians in the subject after 2021. The main reason for this situation is that virtual reality or augmented reality tools are not as widespread as mobile phones or computers in commercial terms. Publications on the subject are mostly in the “Communication” discipline and in the Conference Proceedings Citation Index - (Science (CPCI–S) index. It is expected that the “communication” category will be the category with the highest number of publications due to its relation with journalism. However, the total number of studies in the computer and engineering categories is higher than the communication category. This is due to the studies on the computer-based nature and technical capacities of the tools that provide immersive technologies and their adaptation to different fields.

Secondly, the productive authors, institutions and countries are analyzed. Institution that created the most publications and documents is Shanghai Jiao Tong University with sixteen publications. The countries with the highest number of studies on the subject are the United States of America, the People's Republic of China, Spain, Japan and the United Kingdom. The most cited studies were those originating from the People's Republic of China. It is seen that the institutions and countries where studies on the subject are concentrated are developed countries. The fact that developed countries are pioneers in this issue means that they pay more attention to these high-tech tools and immersive issue. There is no country from the African continent in any list based on the number of articles or citations. In addition to financial constraints in the African continent, the lack of technological infrastructure, differentiation of academic specialisations, limited regional cooperation and access to global research networks may be other factors affecting this result. Yao Liu, who works in the fields of Computer Engineering, Imaging sciences and engineering and telecommunication technologies, is the author with the most publications on the subject.

Thirdly citations, co-citation clusters and collaborations are analyzed. Mel Slater, who works in the field of virtual reality, is the most cited author. The most cited publication on the subject is Mel Slater's article “Enhancing our lives with immersive virtual reality” by Maria V. Sanchez-Vives [27], published in the journal “Frontiers in Robotics and AI”. Mainly since it is a pioneering study in which descriptive concepts on virtual reality are conveyed. In the co-author analysis, Liu, Zhou, Chen, Xiao and Li are the authors who collaborate the most with more than 2 authors per academic study in the field. The studies in the literature were created with the contribution of at least 2 authors. In institutional collaboration, Shanghai Jiao Tong and Aizu are the universities with the most studies, and Stanford and Illinois Universities have established the strongest connection network and the most intensive collaboration. This situation has emerged due to the fact that developed countries can allocate more financial resources to high technology and their academic facilities in both theoretical and practical fields are advanced compared to other institutions and countries.

Fourthly the co-occurrence words and keywords are analyzed. This analysis shows research points and trends for the topic. The most frequently used keywords in the studies were “virtual reality” (286), “360-degree video” (278), “immersive journalism (93) and “Narrative Journalism” (56). Although the studies were handled within the framework of different spelling rules in many different languages or the same words were written differently due to spelling errors, the numbers were reached by counting them in a common cluster. The 360-degree imaging tools and imaging technique required for immersive journalism and the concept of virtual reality constitute the hotspots related to the subject. The association with “narrative journalism” for a more experience-oriented and realistic version of the journalism profession shows a new trend point.

No filter was applied to the studies in the data set of the study and book chapters, editorial materials or conference proceedings in the Web of Science database were also included in the study. During the visualisation of the findings, certain breakpoints were used to make the visuals understandable and detailed.

The study has some limitations. During the search for studies related to the literature, concepts and terms related to the field were added, but it is likely to be associated with terms and concepts that are missing or not yet known. In the future, we can expand search terms or focus on smaller areas of bibliometric analysis. This study refers to the main literature by selecting only publications found in the Web of Science Core collection. In future research, more databases could be used for the analysis. 955 studies constituting the data set of the study were accessed on May 25, 2024. After this date, new articles added to the Web of Science database with the relevant keywords and related to the subject of the study may lead to different findings. Therefore, it is possible that the findings obtained may differ**.**

## 4 Discussion

Journalism is one of the professions directly affected by technology. Rapid developments in digital tools and platforms have fundamentally changed the way news is gathered, reported and consumed. Traditional journalism, which relied heavily on print media and face-to-face interviews, has now transformed into digital journalism using multimedia content, social media and real-time updates.

It is foreseen that the transformation of the journalism profession in the technological context will undergo a change that cannot be evaluated only within the field of social sciences in the coming years and will become a profession that should be handled with a multidisciplinary approach.

This transformation is not only about adopting new tools, but also about reshaping the ethical principles, working models, the value of labor and audience engagement strategies that define journalism.

Therefore, it is recommended that the curriculum in the field of journalism should be revised in parallel with the developments in technology, especially to cover topics in areas such as virtual reality technologies, artificial intelligence and metaverse.

Integrating these topics into journalism education will prepare future journalists to create immersive news experiences, use artificial intelligence for data analysis and content creation, and understand the implications of reporting in virtual environments.

In order to ensure the adaptation of active journalists to technological innovations, it is of great importance to organize training and workshops in cooperation with journalism associations. In this way, journalists can work more effectively and efficiently in a rapidly changing digital world. In addition, such collaborations will encourage the exchange of knowledge and experience among colleagues.

This study is important in terms of listing the studies on “immersive journalism”, which seems inevitable that it will gain importance in the future, considering the changing journalism practices and digital narrative forms that are differentiating day by day, revealing their contexts and guiding the relevant people, especially academics and journalists, on the subject.

## Funding statement

This research did not receive any specific grant from funding agencies in the public, commercial, or not-for-profit sectors.

## Additional information

No additional information is available for this paper.

## Data availability statement

Data will be made available on request.

## CRediT authorship contribution statement

**Mehmet Arif Arık:** Writing – review & editing, Writing – original draft, Visualization. **Murad Karaduman:** Supervision, Methodology, Data curation. **Sibel Karaduman:** Resources, Formal analysis. **Çiğdem Karakaya:** Validation, Formal analysis. **Fulya Erendağ Sümer:** Writing – original draft, Validation, Methodology. **Zuhal Gök Demir:** Writing – review & editing, Supervision, Methodology.

## Declaration of competing interest

The authors declare that they have no known competing financial interests or personal relationships that could have appeared to influence the work reported in this paper.
